# Statistical and Reliability Study on Shear Strength of Recycled Coarse Aggregate Reinforced Concrete Beams

**DOI:** 10.3390/ma14123321

**Published:** 2021-06-16

**Authors:** Hyunjin Ju, Meirzhan Yerzhanov, Alina Serik, Deuckhang Lee, Jong R. Kim

**Affiliations:** 1 Department of Civil and Environmental Engineering, School of Engineering and Digital Sciences, Nazarbayev University, 53 Kabanbay Batyr Avenue, Nur-Sultan 010000, Kazakhstan; hyunjin.ju@nu.edu.kz (H.J.); meirzhan.yerzhanov@nu.edu.kz (M.Y.); alina.serik@nu.edu.kz (A.S.); 2 Department of Architectural Engineering, Chungbuk National University, 1 Chungdae-ro, Seowon-gu, Cheongju, Chungbuk 28644, Korea; dk@cbnu.ac.kr

**Keywords:** recycled aggregate concrete, structural behavior, shear, statistics, reliability

## Abstract

The consumption of structural concrete in the construction industry is rapidly growing, and concrete will remain the main construction material for increasing urbanization all over the world in the near future. Meanwhile, construction and demolition waste from concrete structures is also leading to a significant environmental problem. Therefore, a proper sustainable solution is needed to address this environmental concern. One of the solutions can be using recycled coarse aggregates (RCA) in reinforced concrete (RC) structures. Extensive research has been conducted in this area in recent years. However, the usage of RCA concrete in the industry is still limited due to the absence of structural regulations appropriate to the RCA concrete. This study addresses a safety margin of RCA concrete beams in terms of shear capacity which is comparable to natural coarse aggregates (NCA) concrete beams. To this end, a database for reinforced concrete beams made of recycled coarse aggregates with and without shear reinforcement was established, collecting the shear specimens available from various works in the existing literature. The database was used to statistically identify the strength margin between RCA and NCA concrete beams and to calculate its safety margin based on reliability analysis. Moreover, a comparability study of RCA beams was conducted with its control specimens and with a database for conventional RC beams.

## 1. Introduction

**Figure 1:**
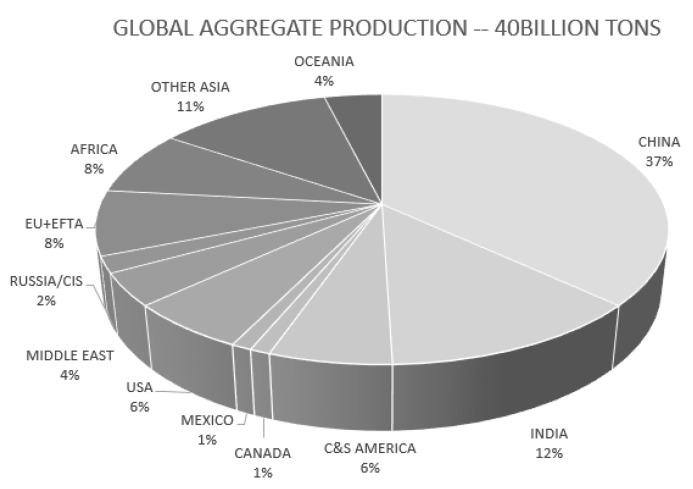
Global aggregate production in 2014 [1].

Concrete is one of the most widely used construction materials, and its demand has been significantly high and will even grow with rapid economic development and urbanization. Concrete mainly consists of coarse aggregates which account for around 70–80% of the volume of the concrete mixture. Thus, the coarse aggregate plays a significant role in the strength and performance of concrete members. Meanwhile, the global demand for new aggregates which are incorporated in the concrete mixture is estimated to exceed 40 billion tons annually as new concrete structures are constructed. Figure 1 shows the distribution of global aggregate demand in countries [1]. Furthermore, an extensive amount of construction and demolition waste (CDW)—about 1.35 billion tons each year in the USA and Europe, and 1.5 billion tons in China—commonly goes to landfills, and it is thus emerging as an environmental concern [2].

Recycling the waste concrete for use as coarse aggregates is a sustainable solution to address the main two environmental concerns related to the depletion of natural aggregates and landfill capacity. However, it has been reported that the presence of old mortar on the surface of the recycled coarse aggregate (RCA) adversely affects its physical and mechanical properties [3,4,5]. The recycled aggregates are produced by crushing the demolished concrete into desirable sizes, and part of the mortar paste from the original concrete remains attached to the surface of the aggregates. Consequently, there are two types of the interfacial transition zone (ITZ) that appear in the concrete made of the RCAs. The one is in RCA itself, which is between the original aggregate and residual mortar, and the other one is between RCA and the new mortar. As a result, it increases the porosity and water absorption in the RCA concrete, while reducing the density of recycled aggregates relative to natural aggregates [6]. Thus, most of the previous studies have mainly focused on the physical properties and treatment methods of recycled aggregates to investigate the material characteristics.

Since the shear failure of reinforced concrete (RC) has a brittle nature, it can be critical unless the concrete member is properly designed against shear considering the material characteristics. Furthermore, the shear resistance of concrete members significantly depends on the aggregate interlocking at the crack surface. Thus, the inferior properties of recycled coarse aggregates negatively affect the aggregate interlock and may reduce the shear resistance [7]. Although comprehensive research has been carried out on the effect of RCA on the shear strength of reinforced concrete beams, the use of recycled aggregate in the industry is still limited due to the lack of design regulations.

Recently, experimental and statistical approaches have been conducted [8,9], and they concluded that the shear strength of RCA concrete beams is comparable to that of normal concrete beams or tends to decrease according to the replacement ratio of RCA. This study is aimed to analyze the experimental data collected from the existing literature available on the shear strength of reinforced concrete beams made of the RCA with/without shear reinforcement. Thus, this study could provide rigorous evidence of the recent research findings and promote the use of RCA concrete beams in terms of shear design. A database on RCA concrete specimens is compared with conventional concrete specimens to investigate the impact of the RCA replacement ratio, compressive strength of concrete, and shear span-to-depth ratio of the beam members. Current design specifications do not consider the use of recycled aggregates to determine the shear capacity of the beam. In this study, the ACI 318–19 provisions for calculating the shear capacity were used to evaluate the shear strengths of RCA concrete beams.

## 2. Shear Database

A database was compiled from available studies on the shear strength of RCA concrete beams with and without shear reinforcement. A review of existing studies revealed that a total of 591 specimens from 41 studies [7,10,11,12,13,14,15,16,17,18,19,20,21,22,23,24,25,26,27,28,29,30,31,32,33,34,35,36,37,38,39,40,41,42,43,44,45,46,47,48,49] were tested and reported between 2001 and 2020. All experimental studies include comparisons of shear strengths of RCA concrete beams with those of natural coarse aggregates (NCA) concrete beams and a key variable is the replacement ratio of RCAs, which ranges from 0% to 100%.

The specimens were rigorously selected from the database through a detailed filtration process to ensure that the experiments were well-executed, and the specimen details are in practical range. Since only slender beams were analyzed in this study, the following selection criteria used by Reineck et al. [50] and Dunkelberg et al. [51] were applied to finalize the shear database of RCA concrete beams: the shear span-to-depth ratio (a/d) equal or greater than 2.4, the compressive strength of concrete ($f_{c}’$) higher than 12 MPa, and the effective width ($b_{w}$) larger than 70 mm. The compressive strength of concrete ($f_{c}’$) in the database is from the original literature where the measured cylindrical compressive strength ($f_{c,cyl}$) is reported. It is well-known that the compressive strength of concrete depends on the sizes and shapes of the samples. The cylinder and cube are the most commonly used specimens to study the compressive strength of concrete. Thus, conversion factors ranging from 0.8 to 0.9 are suggested to convert the cubic compressive strength to cylindrical compressive strength [52]. When the cubic compressive strength ($f_{c,cube}$) is reported in the reference, the conversion factor of 0.8 is multiplied to obtain $f_{c,cyl}$ ($=0.8f_{c,cube}$) for normal strength concrete [53]. To ensure that all specimens failed in shear, the ratio of experimental shear strength to the nominal flexural strength estimated by ACI-318 ($V_{test}/V_{flex}$) was calculated, and the specimens with the ratio equal to or exceeding the ratio of 1.1 were excluded from consideration. In other words, the specimens that may be estimated to have failed in flexure were not considered for estimating the shear capacity of RCA concrete beams. Since this study aims to investigate the effect of recycled coarse aggregates on the shear strength of the reinforced concrete beams, specimens with recycled fine aggregates were also eliminated, which would be a topic of further research. 

**Figure 2:**
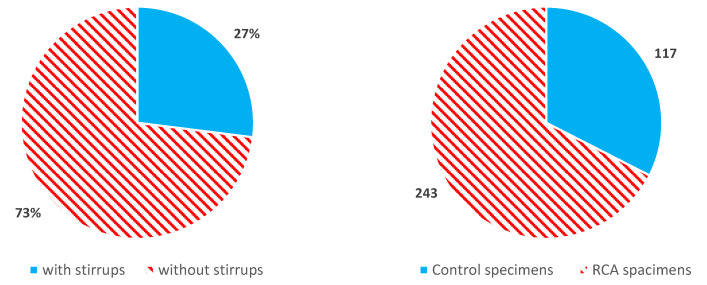
Share of beams with shear reinforcement and control specimens.

**Figure 3:**
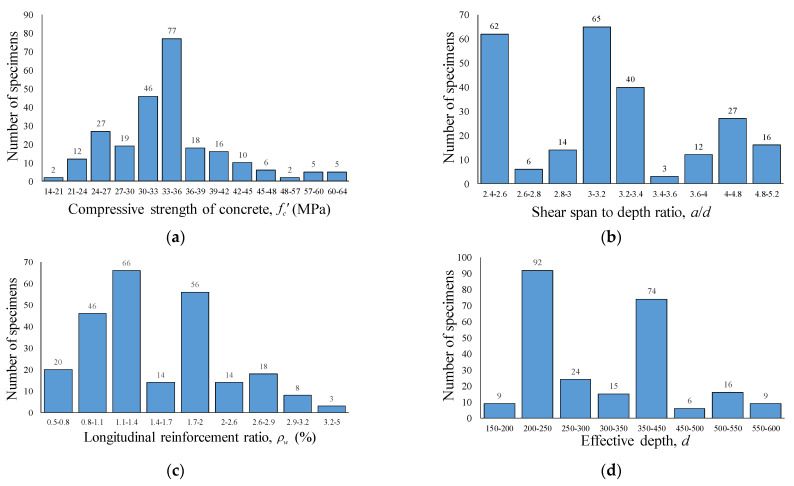
Distribution of key parameters in RCA without shear reinforcement. (**a**) Compressive strength of concrete, MPa. (**b**) Shear span-to-depth ratio. (**c**) Longitudinal reinforcement ratio. (**d**) Effective depth.

Finally, 360 specimens were sorted out among all the collected RCA specimens. The numbers of specimens with and without shear reinforcement are 96 and 264, including control specimens, respectively, as shown in Figure 2. Among them, 117 specimens are control specimens—77 and 40 specimens without and with shear reinforcement, respectively. All specimens were tested under a four-point loading setup. Figure 3 shows the distribution of key parameters of the RCA concrete specimens without shear reinforcement in the database for RCA specimens. In terms of the compressive strength ($f_{c}’$), most of the specimens are in the range of normal strength being concentrated from 24 to 36 MPa, as shown in Figure 3a. The shear span-to-depth ratio (a/d) of specimens without shear reinforcement ranges from 2.4 to 5.1, while most specimens are in the range of 2.4~2.6 and 3.0~3.2. As for the longitudinal reinforcement ratio ($\rho_{w}$), it can be observed from Figure 3c that specimens are mainly distributed in the range of 0.8~2%. Figure 3d shows that the effective depth (d) of beams mostly ranges from 200 to 250 mm and 350 to 400 mm, and the large specimens with an effective depth of 600 mm are also included in the database. 

**Figure 4:**
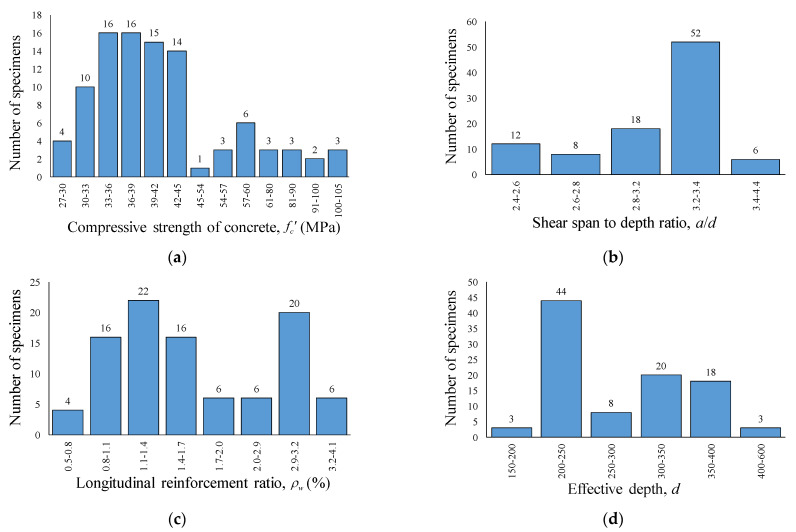
Distribution of key parameters in RCA with shear reinforcement. (**a**) Compressive strength of concrete, MPa. (**b**) Shear span-to-depth ratio. (**c**) Longitudinal reinforcement ratio. (**d**) Effective depth.

Figure 4 shows the distribution of key parameters of RCA concrete specimens with shear reinforcement. The compressive strength of specimens is uniformly distributed in the range of normal strength concrete from 30 to 45 MPa, with some outliers for the high compressive strength of concrete above 60 MPa. The longitudinal reinforcement ratio ranges from 0.75 to 4.09%. A total of 52 test specimens out of 96 in the RCA database with shear reinforcement have the shear span–depth ratio ranging from 3.2 to 3.4. Moreover, almost half of the specimens have an effective depth ranging from 200 to 250 mm, and three specimens had a high effective depth of 600 mm.

## 3. Comparability Study of RCA and NCA Beams

To investigate the implication of recycled coarse aggregates on the shear capacity of reinforced concrete beams, the shear strength ratio between the RCA specimen and its control specimen ($V_{RCA}/V_{NCA}$) is taken into consideration. Several studies [54,55] have claimed that introducing the RCA differently affects shear capacity and mechanical properties of concrete, such as splitting tensile strength, flexural strength, and fracture energy. In addition, the current ACI 318-19 provision suggests the square root of the compressive strength of concrete ($\sqrt{f_{c}’}$) to consider these material properties for calculating the shear capacity of reinforced concrete beams. Therefore, it was decided to use a normalized shear strength ratio, which is expressed as follows:

(1) $$\frac{V_{RCA}}{\sqrt{f_{c}{’}_{\left(RCA\right)}}}/\frac{V_{NCA}}{\sqrt{f_{c}{’}_{\left(NCA\right)}}},$$

where $f_{c}{’}_{\left(RCA\right)}$ and $f_{c}{’}_{\left(NCA\right)}$ are the compressive strength of concrete for RCA and NCA specimens, respectively. The normalized shear strength ratio helps to identify the genuine effect of recycled aggregate on the shear capacity of RC beams. 

The shear equation specified in ACI318-19 for estimating the shear resistance consists of contributions of concrete ($V_{c}$) and shear reinforcement ($V_{s}$). $V_{c}$ is expressed as follows:

(2) $$\begin{array}{rrr} & \;V_{c}=0.66\lambda \lambda_{s}\rho_{w}^{1/3}\sqrt{f_{c}’}b_{w}d & \;\mathrm{for}\;A_{v}<A_{v,\min}\\ \mathrm{either}\;\mathrm{of} & \;V_{c}=0.66\lambda \rho_{w}^{1/3}\sqrt{f_{c}’}b_{w}d\;\mathrm{or}\;V_{c}=0.17\lambda \sqrt{f_{c}’}b_{w}d & \;\mathrm{for}\;A_{v}\geq A_{v,\min}, \end{array}$$

where $b_{w}$ is the web width of member, $A_{v}$ is the area of shear reinforcement within spacing, $A_{v,\min}$ is the minimum area of shear reinforcement within spacing, and $\lambda_{s}$ is the size effect modification factor and equals to $\lambda_{s}=\sqrt{2/\left(1+0.004d\right)}\leq 1$. Moreover, $V_{c}$ shall not be taken greater than $0.42\lambda \sqrt{f_{c}’}b_{w}d$. The shear strength contribution of shear reinforcement ($V_{s}$) is calculated as follows:

(3) $$V_{s}=\frac{A_{v}f_{yv}d}{s},$$

where $f_{yv}$ is the yield strength of shear reinforcement, and s is the spacing of shear reinforcement. Finally, the nominal shear strength ($V_{n}$) of reinforced concrete members is calculated as the sum of the contributions, as follow:

(4) $$V_{n}=V_{c}+V_{s},$$

According to ACI318-19, the maximum allowable $f_{c}’$ is limited up to 69 MPa.

Meanwhile, it was observed that shear capacities reported in papers are fluctuating from approximately 30% reduction to 40% improvement compared to those of control specimens which are NCA concrete beams. These fluctuations may be caused due to different recycling methods, quality of coarse aggregate, concrete mixture, and human and instrumental errors. Moreover, there are some cases where only one experimental datum was available for each replacement ratio in the database, for example, 5%, 23%, 47%, etc. The limited number of tests may lead to the misinterpretation of experimental results, as it is impossible to verify or check human and instrumental errors in these cases. Thus, the specimens were categorized into several groups, according to the replacement ratio to eliminate those uncertainties and differences during quantitative analysis. Table 1 and Table 2 show the grouping of the specimens without and with shear reinforcement, respectively. The groups for specimens without shear reinforcement are for RCA replacement ratio of 1~30%, 31~50%, and 51~100%, while the groups for specimens with shear reinforcement are for 1~50% and 51~100%.

**Figure 5:**
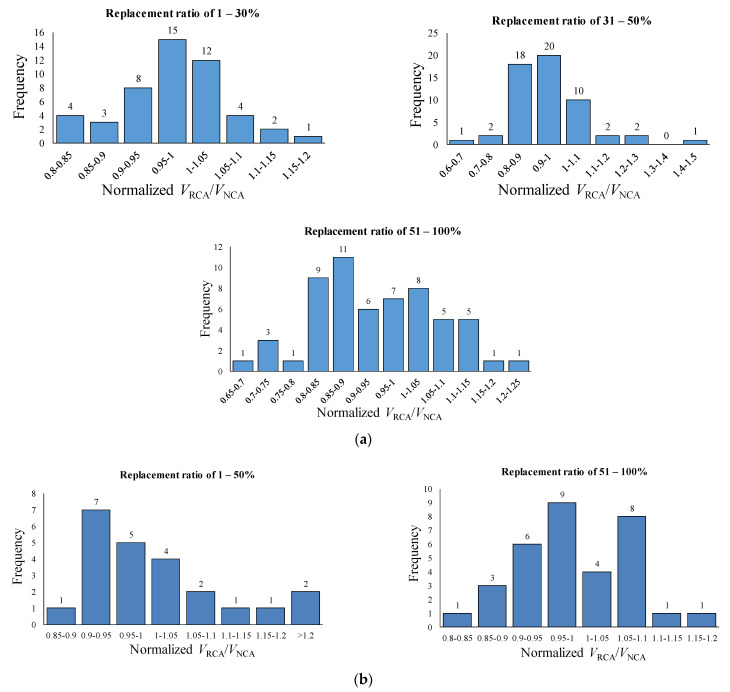
Distribution of normalized ratio in database. (**a**) Specimens without shear reinforcement. (**b**) Specimens with shear reinforcement.

Figure 5 shows the distributions of specimens according to the normalized shear strength ratio. It can be seen that the strength ratio fluctuates above and below unity value, i.e., improvement and reduction in shear capacity. However, all figures are approximately following the normal distribution, where most specimens are concentrated in the middle of the range considered, except for the group of 1–50% for specimens with shear reinforcement. For the group of 1–50%, it is observed that there are many specimens with a strength ratio much higher than the unit value. According to MacGregor [56], a typical coefficient of variance is 0.21 for the shear strength ratio of reinforced concrete beams, which is based on several statistic studies for the shear capacity of the reinforced concrete members. Assuming the control specimens reported in the papers equal to the representative mean for the given section and properties of the beam, most of the specimens in the database are within the range of population standard deviation (σ), except for one specimen from the 31~50% group in the very right tail and eight specimens (three from the 31~50% and five the from 51~100% group) in the left tail for the beams without shear reinforcement. In the case of the beams with shear reinforcement, three specimens (two for 1~50% and one for 51~100% group) in the right tail correspond to the case outside of the standard deviation. Therefore, it can be considered fairly common and as expected results for ordinary reinforced concrete beams with insignificant skew.

**Figure 6:**
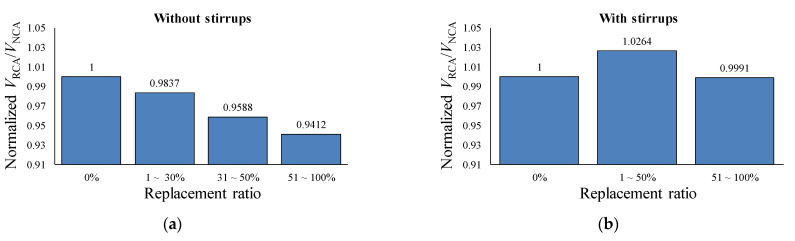
Average normalized ratio for each group. (**a**) Specimens without shear reinforcement. (**b**) Specimens with shear reinforcement.

The mean values were calculated according to the groups of replacement ratio, as presented in Figure 6. For specimens without shear reinforcement, there is a clear trend of reduction in shear capacity as the RCA replacement ratio increases. The reductions in shear capacity by introducing the recycled coarse aggregate are 1.6%, 4.1%, and 5.9% for 1~30%, 31~50%, and 51~100% replacements, respectively. The decrease in shear capacity can be explained by the weak aggregate interlock mechanism. Some amount of binder from the original concrete keeps attached to coarse aggregate after the recycling process. Consequently, an additional interaction layer leads to a decrease in the capacity of aggregate interlocking. For specimens with shear reinforcement, the group 1~50% shows a mean higher than the unit value in the normal shear strength ratio by a little more than 2%, while the group for 51 ~ 100% shows a mean value equal to 0.999. However, for the 1~50% group shown in Figure 5b, the normalized shear strength ratios are concentrated in the range from 0.9 to 1, which takes up 52% (12 specimens out of 23) of the beams. Nevertheless, the higher mean value for group 1~50% was caused by two outliners in the very right tail, which values are 1.28 and 1.43. In the presence of such outliners, the median of the sample is a better measurement tool rather than the mean value. The median of the group for 1~50% is 0.97. Therefore, the decreasing trend in the normalized shear strength ratio of the beams with shear reinforcement is similar to that of the beams without shear reinforcement, while the RCA replacement ratio increases. However, the effect of RCA on the shear capacity of RC beams with shear reinforcement is much less than that of beams without shear reinforcement. It could be explained by the fact that RCA affects only the shear capacity provided by the concrete section, while the contribution of shear reinforcement will remain the same for both RCA and NCA concrete beams. Therefore, the total weight of effect by RCA on the shear capacity of reinforced concrete beams would decrease when shear reinforcement is placed.

**Figure 7:**
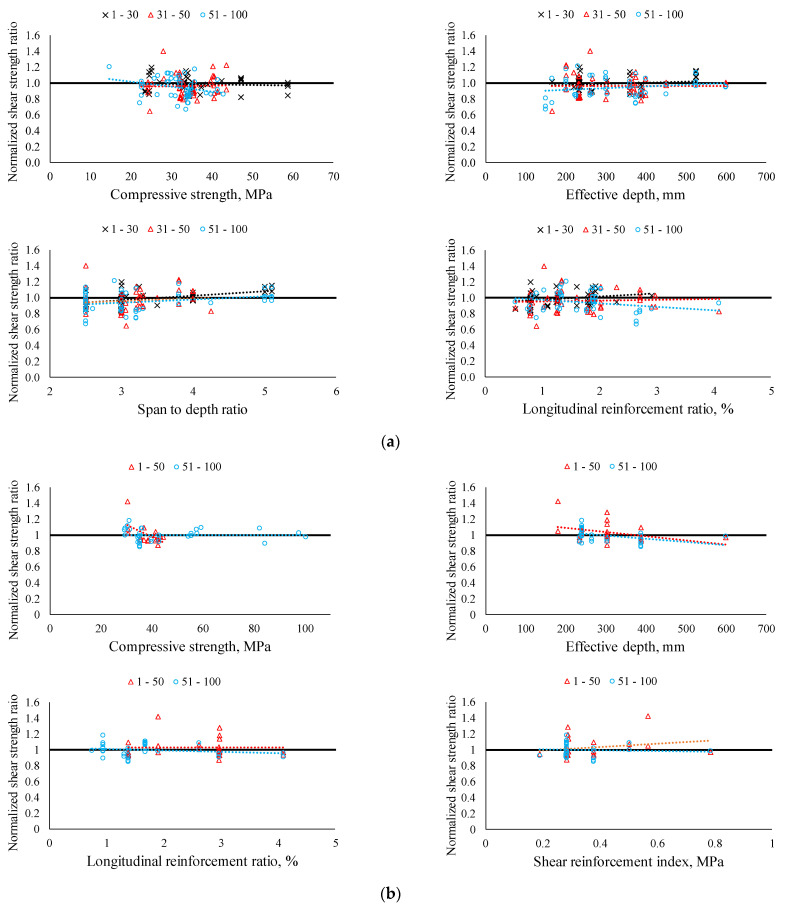
Normalized shear strength ratio against key influencing factors. (**a**) Specimens without shear reinforcement. (**b**) Specimens with shear reinforcement.

Figure 7 shows the normalized shear strength ratios of RCA specimen against key influencing parameters, namely the compressive strength of concrete ($f_{c}’$), the effective depth of the member (d), the span-to-depth ratio (a/d), the longitudinal reinforcement ratio ($\rho_{w}$), and the shear reinforcement index ($\rho_{v}f_{vy}$). For the beams without shear reinforcement, it can be clearly seen that there is no trend in the normalized shear strength ratio according to the key influencing parameters, except the longitudinal reinforcement ratio. The increase in longitudinal reinforcement ratio leads to a decrease in the normalized shear strength ratio of specimens for the RCA replacement ratio of 51~100%. It can be explained by the increase in longitudinal reinforcement ratio which leads to fine shear cracks, and thus the role of aggregate interlock becomes significant. Moreover, specimens with a high replacement ratio have a low modulus of elasticity of concrete compared to control beams made of NCA concrete. Meanwhile, the beams with shear reinforcement showed no clear tendency of normalized shear strength ratio according to key influencing factors, except the effective depth (d). It can be seen that an increase in the effective depth leads to a decrease in normalized shear strength ratio of beams with shear reinforcement for both groups of 1~50% and 51~100%. It should be noted that this tendency cannot be seen in beams without shear reinforcement.

## 4. Statistical Data Analysis

A statistical approach was used to investigate whether there is any statistically significant difference between the recycled coarse aggregate and natural coarse aggregate concrete beams. The normalized shear strength ratio was taken in this analysis, as most of the code provisions use the square root of the compressive strength of concrete ($\sqrt{f_{c}’}$) to calculate the shear strength provided by the concrete section. The paired t-test [57] is a commonly adopted statistical technique applied to evaluate the difference between two population means. This method assumes that the differences between pairs are normally distributed, and in this study, the Shapiro–Wilk test [58], which is a test of normality in statistics, was used to check the normality. As shown above, the calculated means of the normalized shear strength ratio indicated that an increase in the RCA replacement ratio leads to a reduction in the shear capacity of RC beams. Therefore, the following hypothesis was set.

### 4.1. For Beams without Shear Reinforcement

$H_{0}:$Normalized shear strength of beams in the group for 1–30% of RCA replacement ratio is lower than control specimens.

$H_{a}:$Normalized shear strength of the group for 1–30% of RCA replacement ratio larger or equal than control specimens.

RStudio is an integrated development environment for so-called R [59], a programming language for statistical computing and graphics, which was used to conduct the statistical analysis. The analysis result of paired t-test showed a *p*-value of 0.9917. The *p*-value is the probability of rejecting a correct null hypothesis ($H_{0}$). That means, the larger the *p*-value, the more it supports $H_{0}$. The obtained *p*-value is more than the alpha value of 0.05 for the right-tailed test with a confidence level of 95%. This confirms a null hypothesis that the mean of the normalized shear strength of the group for 1~30% is lower than control specimens. The analysis results of the Shapiro–Wilk test showed that differences between pairs can be assumed approximately normally distributed. Moreover, the absence of outliners was confirmed by the Tukey Fence method [60], which is used to detect outliers by identifying detection criteria based on first, third quartiles, and interquartile ranges. The same procedures were conducted for the remaining groups. The *p*-values for these groups for 31~50% and 51~100% were 0.9954 and 0.9985, respectively. From the analysis, it can be concluded that the abovementioned observations about the reduction in shear capacity are statistically confirmed with a 95% confidence level. 

### 4.2. For Beams with Shear Reinforcement

$H_{0}:$Normalized shear strength of the group for 1–50% of RCA replacement ratio equal to control specimens.

$H_{a}:$Normalized shear strength of the group for 1–50% of RCA replacement ratio not equal to control specimens.

The analysis result of paired t-test showed that the *p*-value is 0.7937, which is larger than the alpha value of 0.05. The average population for RCA concrete beams is assumed to be equal to that of NCA concrete beams. In other words, the difference between the averages for RCA and NCA is not large enough to be statistically significant. The analysis results of the Shapiro–Wilk test showed that differences between pairs cannot be assumed as a normal distribution with a moderate violation; that is, the violation of normality assumption is not that significant. Moreover, the paired t-test is considered robust for moderate violation of the normality assumption. The *p*-value for the group for 51~100% was 0.8412. From the analysis, it can be concluded that the average population of RCA and NCA concrete beams can be statistically considered equal with a 95% confidence level.

## 5. Reliability Analysis

Although the introduction of the recycled coarse aggregate leads to a reduction in the shear capacity of reinforced concrete beams, only an average 6% reduction may not have a significant effect on design safety when considering the inherent uncertainties in the shear mechanism. In addition, the code provisions are conservatively established to estimate the shear capacity of the RC beams. Thus, a reliability analysis was conducted to check this point and proposed an appropriate strength reduction factor for the shear design of RCA concrete beams if required. 

To investigate whether a new reduction factor should be introduced in designing RCA concrete beams against shear, the reliability index (β) was estimated for all collected specimens in the database. The estimation was based on the first-order reliability method (FORM). There are various advanced and practical techniques related to reliability which are suitable for a highly non-linear problem and a system-based analysis. In this study, FORM is quite feasible and even computationally economical and efficient since it is used for a member-based analysis, especially the shear strength rather than structural behavior. The load combination was chosen to be 1.2D+1.6L, and the safety factor (SF) is defined as follows:

(5) $$SF=\frac{(1.2D+1.6L)/\varphi }{D+L},$$

where D is dead load, L is live load, and φ is the strength reduction factor. Then, the limit state function (g) is expressed as follows:

(6) $$g=SF\times V_{n}-V_{test},$$

where $V_{n}$ is the nominal shear capacity calculated by ACI 318-19 of Equation (4), and $V_{test}$ is the shear strength obtained from the test data. As the shear equation specified in ACI 318-19 consists of random variables, the bias factors and coefficient of variances (COVs) were taken from Nowak and Szerszen’s study [61], as presented in Table 3. For the compressive strength of concrete, which has several bias factors depending on its nominal value, the closest upper value in Table 3 was taken.

The FORM analysis was carried out by utilizing the probability distribution of the random variables which are used to calculate the nominal shear strength and the limit state function. Based on these calculations, the reliability index was identified for all the beams in the database. The first-order form of limit state function can be written as follows:

(7) $$g(X_{1},X_{2},\ldots ,X_{n})=\sum_{i=1}^{n}(X_{i}’-x_{i}’)\left(\frac{\partial g}{\partial X_{i}’}\right),$$

where $X_{i}$ is the random variable and $x_{i}$ is the random variable at the most probable failure point. $X_{i}’$ and $x_{i}’$ are the variance-normalized values and calculated as follows:

(8) $$X_{i}’=\frac{X_{i}-\mu_{X_{i}}}{\sigma_{X_{i}}}$$

(9) $$x_{i}’=\frac{x_{i}-\mu_{X_{i}}}{\sigma_{X_{i}}},$$

where $\mu_{X_{i}}$ is the mean value of a variable and $\sigma_{X_{i}}$ is the standard deviation of the variable.

The reliability index (β) is taken as a ratio of mean to the variance of limit state function. At the same time, the mean and variance of limit state function can be calculated by the first-order approximation, as follows:

(10) $$\beta =\frac{\mu_{g}}{\sigma_{g}}=\frac{-\sum_{i=1}^{n}x_{i}’(\partial g/\partial X_{i}’)}{\sqrt{\sum_{i=1}^{n}{\left(\partial g/\partial X_{i}’\right)}^{2}}},$$

**Figure 8:**
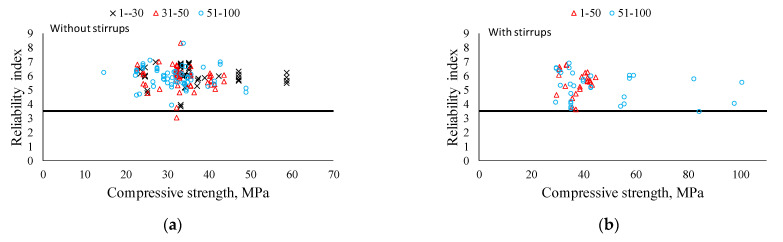
Reliability index values for RCA beams. (**a**) Without shear reinforcement. (**b**) With shear reinforcement.

Figure 8 shows the calculated reliability index for specimens in the database with and without shear reinforcement. For the shear equation of ACI 318-19, the strength reduction factor of 0.75 was used and the live load was taken equal to dead load [62]. It should be mentioned that ACI 318-19 targets the reliability index of 3.5, which was proposed by Nowak and Szerszen [61]. It can be seen that the reliability indices for almost all specimens are above 3.5, except for only one specimen in the database without shear reinforcement. Moreover, it should be mentioned that distributions of the reliability indices are almost the same for all groups. Therefore, it can be concluded that the safety level of RCA concrete beams in terms of the shear design by ACI 318-19 code provision is at an acceptable level for any replacement ratios. In addition, the reduction in shear capacity for beams without shear reinforcement does not significantly affect the conservativeness of the current equation, and the reliability index for the beams with shear reinforcement substantiates its safety level in addition to the results of the statistical analysis above. 

## 6. Comparability Study of RCA with RC Database

In this section, the shear strengths of RCA concrete beams are compared with those of RC beams from a well-established database. The RC database was taken from Yerzhanov and Lee’s study [63], and it consists of test results for 954 RC beams. From these experimental results, the number of RC specimens without and with shear reinforcement are 784 and 170, respectively. Four key parameters that affect concrete contribution to shear strength are taken into account, namely the compressive strength of concrete (d), the effective depth of the member ($f_{c}’$), span-to-depth ratio (a/d), longitudinal reinforcement ratio ($\rho_{w}$), and shear reinforcement index ($\rho_{v}f_{vy}$).

**Figure 9:**
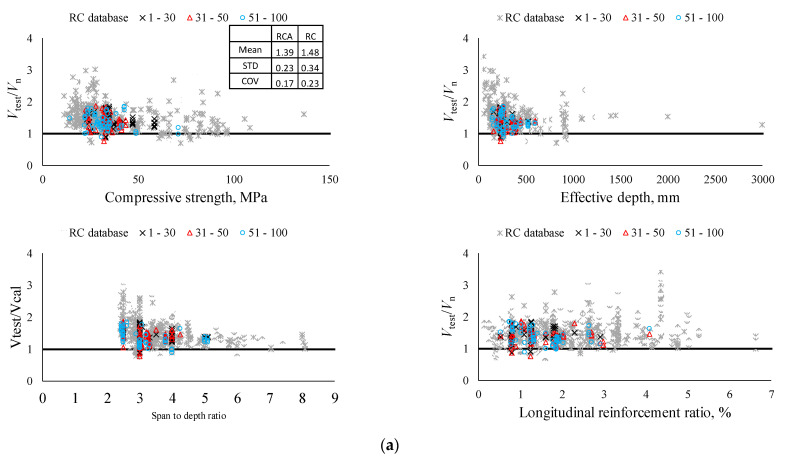

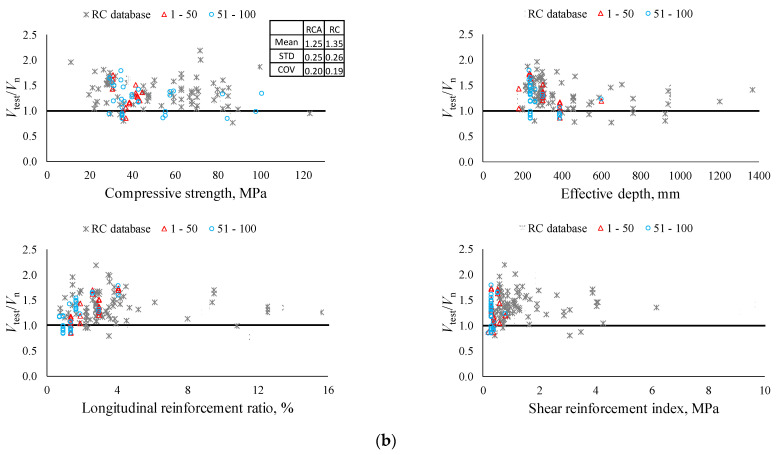
Verification of RCA beam results with RC beam database. (**a**) Specimens without shear reinforcement. (**b**) Specimens with shear reinforcement.

Figure 9 shows the shear strength ratio ($V_{test}/V_{n}$) against abovementioned key parameters for both databases of RCA concrete beams and RC concrete beams, where $V_{test}$ and $V_{n}$ are the shear strengths obtained from test results and calculated by ACI 318-19, respectively. For beams without shear reinforcement, the RCA concrete beams fall within the central portion of the RC database, and they follow the same general trend. The overall mean value of $V_{test}/V_{n}$ for the RCA database is expectedly smaller than that for the RC database. Furthermore, the mean difference between RCA and RC databases is 6.1%, which is close to values obtained from comparing RCA concrete beams with control specimens above. It should be noted that the COV of the RCA database is quite smaller than that of the RC database. The RC database contains a wide range of key parameters to identify the size effect, the effect of high-strength concrete, etc., which cause fluctuations in shear strength. For beams with shear reinforcement, the same conclusion can be drawn with the same general trend as RC beams can be found.

**Figure 10:**
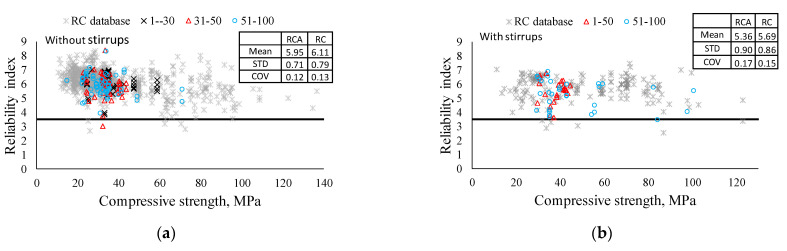
Reliability indices of RCA beams with RC database. (**a**) Specimens without shear reinforcement. (**b**) Specimens with shear reinforcement.

Figure 10 presents the reliability indices (β) for RCA and RC databases. RCA concrete beams follow the general trend given for the RC database, and it is confirmed that the mean β values of RCA concrete beams are a bit less than those of RC beams. Thus, it can be concluded that the current shear design equation can be applied without any adverse effect on safety and conservativeness in the shear design of RCA concrete beams.

## 7. Discussion

For beams without shear reinforcement, a comparability study of RCA and NCA beams showed that the introduction of recycled coarse aggregate will result in shear capacity reduction. The same result was shown in statistical analysis, which stated that these reductions are statistically significant. However, the values of these reductions are only 1.6%, 4.1%, and 5.9% for 1~30%, 31~50%, and 51~100% replacement ratios, respectively. Therefore, it was assumed that these small fluctuations will be negligible in terms of member safety. To check it, a reliability analysis was conducted. It showed that the mean reliability index is 5.95, which is higher than the ACI target reliability index. Moreover, RCA performance was compared with RC. The comparison showed that there is no difference between them in terms of strength capacity, as well as reliability index. It can be concluded that the clear reduction in shear capacity can be neglected due to its small value and conservativeness of the current ACI equation. Therefore, we propose using the same equation for RC and RCA beams. There is a clearer picture for beams with shear reinforcement. Comparability study of RCA and NCA beams showed mean values are quite close to unity, 2.6% increase and 0.1% reduction for 1~50% and 51~100% replacements, respectively. Statistical analysis also showed that the difference is not statistically significant. A comparability study between RC and RCA confirmed that there is no considerable difference in shear capacity. It can be explained that coarse aggregate only affects the shear resistance of concrete. Therefore, the effect of recycled coarse aggregate becomes negligible.

## 8. Conclusions

In this study, the performance of RCA concrete beams was assessed regarding its shear capacity, for which an extensive literature review was conducted and an up-to-date database for RCA concrete beams was established. The analysis of the established database allowed us to draw the following conclusions:

1. The comparability study of RCA concrete beams with its control specimens showed that there is a small reduction in the shear capacity of RCA concrete beams. Moreover, the capacity reduction grows as the replacement ratio increases, and it was much less for the beams with shear reinforcement.
2. For beams without shear reinforcement, a paired t-test confirmed the difference between RCA concrete specimens and its control specimens. It was statistically proven that the average shear capacity of RCA beams is lower than that of NCA beams. On the other hand, the paired t-test showed that there is no statistically significant difference between RCA and NCA concrete beams with shear reinforcement.
3. The reliability analysis showed that the shear strength equation of current ACI 318-19 can be used for the shear design of RCA beams with a desirable safety level.
4. A comparability study with well-established RC beams indicated that there is no difference in shear capacity between RCA and RC databases. Moreover, RCA concrete beams follow general trends and safety margins, which are set for conventional reinforced concrete members, regardless of key influencing factors or reliability index.
5. This study only focused on the shear strength of RCA concrete beams, not structural behavior. Thus, the structural performance of the RCA concrete beams could be further studied in terms of shear behavior related to stiffness, ductility, and even durability.

## Figures and Tables

**Table 1:** Grouping of specimens without shear reinforcement according to RCA replacement ratio.

RCA Replacement Ratio (%)	Normalized Shear Strength Ratio (Equation (1))	Total Number	Group (%)	Normalized Shear Strength Ratio for the Group	Total Number
5	0.984	1	1~30	0.984	54
10	0.889	2			
15	0.989	17			
16	0.973	1			
20	0.892	2			
22	1.029	1			
23	0.852	1			
25	0.896	2			
30	1.004	27			
35	0.820	2	31~50	0.959	60
40	1.131	1			
47	0.892	1			
50	0.962	56			
60	0.984	4	51~100	0.941	59 (73) *
70	1.058	1			
75	0.896	1			
100	0.938	67			

* 14 specimens with 100% replacement were not counted, as there is no information on control specimens.

**Table 2:** Grouping of specimens with shear reinforcement according to RCA replacement ratio.

RCA Replacement Ratio (%)	Normalized Shear Strength Ratio (Equation (1))	Total Number	Group (%)	Normalized Shear Strength Ratio for the Group	Total Number
20	0.968	6	1~50	1.026	23
22	0.910	2			
25	1.426	1			
40	1.044	2			
47	1.021	2			
50	1.042	10			
100	0.999	33	51~100	0.996	33

**Table 3:** Statistical parameter for variables [62].

Parameters	Nominal Value	Bias Factor	COV
$f_{c}’$	48	1.19	0.115
	55	1.09	0.090
	62	1.16	0.100
	69	1.13	0.115
	83	1.04	0.105
$b_{w}$	–	1.00	0.060
d	–	1.00	0.060
$dia_{long.rein.}$	–	1.00	0.060
$f_{vy}$	–	1.145	0.050
s	–	1.00	0.060
$dia_{trans.rein.}$	–	1.00	0.060
RC beam, shear	–	1.16	0.120

## Data Availability

The data presented in this study are available on request from the corresponding author.
